# Revisiting Inbred Mouse Models to Study the Developing Brain: The Potential Role of Intestinal Microbiota

**DOI:** 10.3389/fnhum.2018.00358

**Published:** 2018-09-19

**Authors:** Reinaldo B. Oriá, João O. Malva, Patricia L. Foley, Raul S. Freitas, David T. Bolick, Richard L. Guerrant

**Affiliations:** ^1^Laboratory of Tissue Healing, Ontogeny and Nutrition, Department of Morphology and the Institute of Biomedicine, School of Medicine, Federal University of Ceara, Fortaleza, Brazil; ^2^Coimbra Institute for Clinical and Biomedical Research (iCBR), Faculty of Medicine, University of Coimbra, Coimbra, Portugal; ^3^Division of Comparative Medicine, Department of Microbiology and Immunology, Georgetown University, Washington, DC, United States; ^4^Division of Infectious Diseases and International Health, Center for Global Health, University of Virginia, Charlottesville, VA, United States

**Keywords:** mouse models, developing brain, immune system, environment, intestinal microbiota

The life-long cumulative exposures (exposome) to environmental contaminants (even low-grade lead, mercury, arsenic etc.) and biological hazards (favoring enteric pathogens and altered “unhealthy” intestinal microbiota) alone or in combination are now being increasingly recognized to deleteriously influence the brain's development and potentially the way the brain copes with aging-related conditions, including neurodegenerative diseases (Costa et al., 2004; Senut et al., 2012; Tshala-Katumbay et al., 2015). The latter may involve sub-optimal development of “cognitive reserve,” which is likely dependent upon a “healthy” and enriched environment to which one is exposed early in life. The potential importance of cognitive reserves to protect from aging-related neurodegeneration is suggested by post-mortem evidence showing that some individuals are better adapted to Alzheimer's disease (AD) related brain injury than others (Marques et al., 2016); some patients who show post-mortem beta-amyloid plaques in the brain had not suffered from AD symptoms during life.

The early life intestinal microbiome is now being acknowledged as a determinant factor influencing human behavior (Oriá et al., 2016; Lima-Ojeda et al., 2017; Carlson et al., 2018) and for immune system maturation (Mulder et al., 2011; Olszak et al., 2012; Nash et al., 2017). If chronically disrupted it may even predispose individuals to neuropsychiatric diseases later in life (Petra et al., 2015; Lima-Ojeda et al., 2017). In addition, in the first 2 years of life, a critical period when key processes toward brain maturation such as myelination and synaptogenesis occur, the intestinal microbiota have not yet reached full “adulthood” maturation (Fanaro et al., 2003; Olivares et al., 2018). As these biological processes overlap in this critical period, environmental challenges early in life, such as enteric diseases, malnutrition and altered maturation of the intestinal microbiota may jeopardize the cognitive development and cause later life metabolic dysfunction in these children (DeBoer et al., 2012; Guerrant et al., 2013). However in spite of the potential implications of early-life microbiota colonization to life-long health, animal models (such as germ-free and antibiotic treatment) widely used to study causal relationship with gut microbiota have limitations in how they affect the brain, and in their ability to model human conditions where environmental conditions are dynamic and constantly changing. Furthermore, the use of mouse models to research into microbiome-brain-immune should be taken with caution, since the species composition of the gut microbiota (and its regulation) can be fairly dissimilar in distinct mammalian taxa (Ericsson and Franklin, 2015; Laukens et al., 2016), therefore findings from mouse gut research may not be extrapolated to humans. In addition, reproducibility of findings from animal models may be affected by the intestinal microbiota composition that may vary with animal housing, including type of caging, type and frequency of bedding change and water source. In addition, animals from different vendors may have distinct intestinal microbiota characteristics (Ericsson and Franklin, 2015).

Animal models to study the brain still rely too much on standard “clean-barrier” or even “enriched” vivarium environments for reproduction of the data, however such models may truly miss the mark by not accurately reproducing the “realistic” environment of early life for most of the world's populations (such as in impoverished settings of the developing world). As the brain is a biological sensor, changes in the environment are an essential determinant of structural and functional neural modifications to optimize species survival; including intergenerational transfer of biological information as a genetic imprint to the offspring. This is a key factor in order to successfully pass (genetically or epigenetically) the “environmentally-fit” genes to other generations.

The use of laboratory mice in neuroscience studies (and in basic science in general) have slowly switched over the past four decades from outbred stocks to almost exclusive use of inbred strains, with C57BL/6 strains now the predominant rodent model. As a modern biological research tool three important needs have driven this transition: (1) the need for genetically-engineered mice to understand the role of genes regulating biological phenomena by knocking-down or knocking-in (e.g., targeted replacement) techniques, which have required an inbred background; (2) The need to control for mouse genetic variability (seen in outbred mice), which could mask the “true” phenotype determined by the experimental condition in a controlled-manner; and (3) The reproducibility of the findings by other research groups, which is a fundamental caveat of the scientific method. This has also driven the housing and care of mouse models toward more controlled and pristine conditions and the pursuit of laboratory animal housing standardization and specific pathogen free (SPF), barrier-style vivaria for pre-clinical research. These facilities were designed to prevent potential effects of widely varying environmental conditions, microbial status, and chemical contaminants. Technologies rapidly developed to maintain mice in almost sterile conditions including HEPA filtered ventilated housing racks, HEPA filtered changing stations, autoclaved cages, autoclaved or irradiated feed, and drinking water treated in some fashion (reverse osmosis, autoclaving, mechanical filtration, chlorination, and/or acidification); all with the intent to exclude a range of microbial agents and other environmental contaminants thought to possibly “interfere” with animal health and research outcomes.

As the role of the intestinal-brain axis arises as a key factor in regulating brain responses to the environment, understanding how exposure to contaminated or “unhealthy” environments (fecal coliforms, enteric pathogens, heavy metals, imbalanced diets etc.) can affect the intestinal microbiome in the first years of life becomes critical to define and understand. The intestinal microbiome is considered “plastic” early in life (first 2 years of post-natal life). Maturation of the intestinal microbiota may be delayed if the host animal is exposed to dietary limitations or to contaminated environments (Subramanian et al., 2014). The disrupted intestinal microbiota (with small intestinal bacterial overgrowth and dysbiosis) early in life due to dietary limitations or contaminated environments (Donowitz and Petri, 2015; Donowitz et al., 2016) could have a key role in affecting brain development at the same time when the brain also has its greatest plasticity (Clarke et al., 2013). The way the environmental-born intestinal microbiota may drive the immune system and associated cognitive outcomes can be depicted by findings that weanling germ-free mice have increased serum IgE levels, which rely on upregulated ThCD4-derived IL-4 rather than the IgE baseline levels seen in mice under pathogen-free vivaria (Cahenzli et al., 2013). It has been recognized that meningeal ThCD-4-derived IL-4 has been associated with pro-cognitive effects in mice (Derecki et al., 2010). Interestingly, germ free mice show improved motor activity and reduced anxiety, compared with SPF mice with a normal gut microbiota (Diaz et al., 2011; Neufeld et al., 2011), however, with poor social development (Desbonnet et al., 2014) and with brain neurochemical changes (Clarke et al., 2013). On the other hand, intestinal microbial dysbiosis induced by antibiotic treatment has been shown to impair novel object recognition in mice (Fröhlich et al., 2016), and was associated with decreased hippocampal transcriptions levels of NPY1R, NPY2R, BDNF, and NMDAR2B (GRIN2B), well-known modulators of hippocampal glutamatergic transmission, therefore suggesting an association between gut microbiota alterations and novel object recognition impairment.

Microbiota dysbiosis and intestinal barrier dysfunction induced by chronic inflammation may lead to circulating endotoxins, such as LPS, that may cause long-term disruption in the cognitive reserve relying on newly generated neurons from the hippocampus in adulthood (Valero et al., 2014). Nevertheless, neonatal peripheral injection of LPS may lead to priming of brain microglia with a second challenge during adulthood causing impaired neurogenesis and poor spatial memory (Dinel et al., 2014). Of note, microglia function may be influenced by microbiota-derived short-chain fatty acids (Erny et al., 2015). Limited microbiota complexity also resulted in defective microglia (Erny et al., 2015, 2017). The intestinal microbiota composition may also influence the blood-brain barrier (BBB) permeability (Braniste et al., 2014; Labzin et al., 2018). This is an important issue, as microbiota dysbiosis may affect innate immune responses. Such effects may be exemplified by the known induction of Th17 cells by segmented filamentous bacteria (Gaboriau-Routhiau et al., 2009) (found in rodent microbiota but not humans) and *Bacteroides fragilis-*derived polysaccharide A induction of Treg cells (Mazmanian et al., 2005). Another intriguing aspect is that the gut microbiota driven by western diets might favor the production of autoantibodies with implications for systemic inflammation and neurodegenerative diseases (Petta et al., 2018).

The exposure to early intestinal microbial products may lastingly affect the BBB permeability, a key factor in regulating the brain milieu and function. It has been shown that germ free mice have more permeable BBB with decreased occludin and claudin-5 levels than their pathogen-free raised counterparts (Braniste et al., 2014; Sharon et al., 2016). Few studies have addressed the BBB following enteric infections and malnutrition in young mice, although bacterial infections following acute stress have been associated with memory impairment (Gareau et al., 2011). The brain ultrastructure changes in germ-free mice also include increased hippocampal neurogenesis (Ogbonnaya et al., 2015), as well as decreased microglial maturation and ramification (Erny et al., 2015), and increased prefrontal cortex myelination (Hoban et al., 2016). Another interesting observation is that laboratory mice do not show effector-differentiated mucosal memory T-cells and therefore do not fully resemble the immune systems of adult humans (Beura et al., 2016).

In addition, the concept that the brain is considered an “immune privileged” site, is now shaped by the exciting discovery of lymphatic vessel in the dura sinus and the trafficking of immune cells between the brain and the deep cervical lymph nodes, which may shed light on the potential role of the adaptive immune system surveillance in response to intestinal microbial population changes in homeostasis and disease and the signals to the central nervous system (Louveau et al., 2015; Kipnis and Filiano, 2018), perhaps with the engagement of the meningeal immune system (Kipnis, 2016) as well during the course of a life span.

As new gut-brain research emerges and evolves to unravel the key effects of intestinal microbiota on brain development, there is a need to revisit animal modeling and housing that can better simulate the impoverished environments faced by many people around the world, to identify and address brain-related markers and outcomes. The understanding of this premise is a critical first step for neuroscientists to rethink laboratory housing standardization in some research initiatives in modeling brain development studies. Defining laboratory housing conditions in order to evaluate specific dietary, enteric pathogen and microbiome effects relevant to “real-life” conditions, including such problems as “environmental enteropathy” is challenging, with a need to preserve what has been accomplished by the modern vivarium and environmental standardization, which favors research quality and reproducibility.

One example of this environmental issue can be depicted by murine model studies of malnutrition that have been conducted in the standard barrier-protected vivarium, using inbred mouse models (without environmentally contaminated-intestinal microbiota) and looked at brain markers. Studies that assume no environmental contamination may have relevance to populations in rich-resource developed countries with a high quality of life and good public health systems, where in general the level of environment contamination is low. However they are much less likely to be relevant to the majority of undernourished people residing in sub-Saharan Africa, Latin America, and Asia where poverty is still a huge public health problem and fecally-contaminated environments prevail exposing children to enteric infections and malnutrition. The condition called *environmental enteropathy* has been described in children to define a chronic subclinical intestinal inflammation, mediated by a T-cell response, a cause or consequence of malnutrition and enteric infections (even without diarrhea) in the first years of life (Korpe and Petri, 2012). This malnutrition-enteric infections cycle has been associated with poor cognitive and metabolic outcomes that may be sustained well into late childhood or even later life (Guerrant et al., 2013).

Early enteric infections and systemic inflammation may profoundly affect neurodevelopment in children (Donowitz et al., 2018). Initial work to address these problems of dissecting diet, microbiome, and pathogen effects in carefully defined murine models is underway. These include studies of diets, antibiotics and pathogen effects, and demonstrate the importance of recognizing the complexity of these often quite specific interactions. Examples include recent metabolic studies of protein and zinc deficient diets (Mayneris-Perxachs et al., 2016) as well as effects of specific pathogens including *Cryptosporidium, Giardia, enteroaggregative E. coli* and others (Coutinho et al., 2008; Barash et al., 2017; Bartelt et al., 2017). Hence, application of the increasingly available tools of genetics (including knockdown and targeted transgenic murine models as well as GWAS, SNPchip and epigenetic methylation studies), microbiology (including deep sequencing of the microbiota, “germ free,” “humanized” as well as TAC card diagnostics) (Liu et al., 2013) and especially now metabolomics (Farras et al., 2018), selective cell sorting and signaling and localization of transcriptomics in the gut and CNS, (Hoban et al., 2016; Zubcevic et al., 2017) are critical to understand the acute and long-term impact of widespread enteropathy on cognitive/brain development and maturation; as well as to designing and assessing the effects of targeted safe and effective interventions to ameliorate these devastating effects and optimize healthy cognition/brain development and immune system maturation.

We believe that there is no single and definitive response to assess the role played by environmental contaminants on gut microbiota and health outcomes in the controlled vivarium; the solutions may depend on specific research questions and on the environmental particularities of each animal setting. Recently, efforts have been made to reconstitute the intestinal microbiota from the laboratory mouse living in standard pathogen-free conditions with a more “natural” microbiota obtained from wild mice. Such efforts of microbiota transfer from wild mice have led to reduced inflammation and improved resistance to infectious pathogens in the laboratory mice (Rosshart et al., 2017). It would be interesting to know whether such transfer could improve behavior following malnutrition and enteric infections in that host in the most sensitive times of early post-natal cognitive development. Another good approach is suggested by Shin et al. (2018), who compared intestinal microbiome in young and aged mice and utilized a cage switch protocol to promote exchange of microbiota from these different populations of mice under standard controlled conditions.

In conclusion, the intestinal microbiota is dynamic in the first years of life, when its constitution is profoundly influenced by the surrounding environment. Early life intestinal microbiota is therefore considered immature and more “plastic” until it approaches adult-like characteristics in later childhood. The brain is also very plastic in the first 2 years of life, the very same time period when children may be exposed to changing diets and contaminated environments, and afflicted with clinical and subclinical enteric infections. Enteric infections in the first 2 years of life may alter the normal progression of the intestinal microbiota to “adult-like” maturation and may disrupt optimal brain development, especially in children living in impoverished settings who likely receive a heavier and prolonged infection load. Neuroscience research has relied on inbred mouse models and barrier-protected housing conditions where mice are kept in “clean” standardized environments to study the biological phenomena, and secure conditions that favor the reproducibility of data. In this opinion paper, we suggest the need to revisit animal models and housing conditions to study early life brain development, with greater emphasis on modeling contaminated environments, higher pathogen load, altered microbiota and environmental enteropathy (EE) and better represent conditions that afflict many children living in impoverished communities around the world. Such innovative brain research can elucidate mechanisms of brain metabolism and cognitive function at both extremes of age and potential innovative interventions to ameliorate their disruption.

## Author contributions

All authors listed have made a substantial, direct and intellectual contribution to the work, and approved it for publication.

### Conflict of interest statement

The authors declare that the research was conducted in the absence of any commercial or financial relationships that could be construed as a potential conflict of interest. The handling Editor declared a shared affiliation, though no other collaboration, with some of the authors DB, RG.
